# Clinical Significance and Biological Role of HuR in Head and Neck Carcinomas

**DOI:** 10.1155/2018/4020937

**Published:** 2018-01-28

**Authors:** Georgia Levidou, Ioly Kotta-Loizou, Jason Tasoulas, Thomas Papadopoulos, Stamatios Theocharis

**Affiliations:** ^1^First Department of Pathology, Medical School, National and Kapodistrian University of Athens, Athens, Greece; ^2^Department of Pathology, Klinikum Nuremberg, Paracelsus Medical University, Nuremberg, Germany; ^3^Department of Life Sciences, Faculty of Natural Sciences, Imperial College London, London, UK

## Abstract

**Background:**

Hu-antigen R (HuR) is a posttranscriptional regulator of several target mRNAs, implicated in carcinogenesis. This review aims to present the current evidence regarding the biological role and potential clinical significance of HuR in head and neck carcinomas.

**Methods:**

The existing literature concerning HuR expression and function in head and neck carcinomas is critically presented and summarised.

**Results:**

HuR is expressed in the majority of the examined samples, showing higher cytoplasmic levels in malignant or premalignant cases. Moreover, HuR modulates several genes implicated in biological processes important for malignant transformation, growth, and invasiveness. HuR seems to be an adverse prognosticator in patients with OSCCs, whereas a correlation with a more aggressive phenotype is reported in several types of carcinomas.

**Conclusions:**

A consistent role of HuR in the carcinogenesis and progression of head and neck carcinomas is suggested; nevertheless, further studies are warranted to expand the present information.

## 1. Introduction

Accumulating evidence attributes a critical role to posttranscriptional regulation of gene expression, mediated by RNA-binding proteins (RBPs), in human disease and particularly malignant transformation [1]. This is not surprising since many important cellular processes, such as proliferation, differentiation, and apoptosis, are reportedly regulated at posttranscriptional level [2]. In fact, RBPs associate with the 3′ untranslated region of the target mRNAs and thus can regulate all phases of RNA biogenesis, including splicing, capping, 3′ end formation, subcellular localisation, translation, and finally degradation [3].

One well-characterised posttranscriptional regulator is the HuR protein, a member of embryonic lethal abnormal vision Drosophila-like family (ELAV) of RBPs, consisting of Hel-N1/HuB, HuC, HuD, and HuR proteins, initially identified as specific tumour antigens in patients with paraneoplastic neurological phenomena [4, 5]. HuR protein is normally expressed in a variety of cell types, including adipose tissue and the intestine, spleen, thymus, and testis with low-level expression in the liver and uterus [6, 7].

HuR is implicated in the regulation of the expression of many genes, and the alteration of its protein levels or its localisation has been associated with numerous human diseases, such as pathologic inflammation, atherosclerosis, or ischaemia [8–10]. Moreover, many transcripts coding for factors involved in carcinogenesis, including oncogenes, growth, and antiapoptotic factors, are described among HuR targets [11, 12]. Among these, HuR has an important role in tumoural angiogenesis [13]. Thus, it is not unexpected that an aberrant overexpression of HuR has been repeatedly associated with malignant transformation and increased nuclear and/or cytoplasmic HuR expression is correlated with patient prognosis in a significant number of human malignancies, such as lung adenocarcinoma, gallbladder carcinoma, urothelial carcinoma, ovarian cancer, breast cancer, and colon cancer [11].

Head and neck tumours constitute the eighth leading causes of cancer-related death worldwide, having an incidence which varies among different geographic areas and is significantly higher in developing countries when compared to the European Union and North America, probably due to higher tobacco use and alcohol consumption habits and the lower socioeconomic status in these areas [14]. They encompass a highly complex and heterogenous group of tumour types, arising from different cell progenitors and anatomic sites. Although more than 90% of the cases are of the same histological type, namely, squamous cell carcinoma, even among these, a degree of diversification is noted, with respect to risk factors, pathogenesis, and finally clinical behaviour [15–20]. For example, squamous cell carcinoma of the oropharynx can be broadly divided into HPV^+^ and HPV^−^ cases, types driven by completely different pathophysiological mechanisms [19]. Interestingly, recent studies have shown that HuR knockdown attenuates the oncogenic potential of oral cancer cells [21], whereas a number of studies implicate HuR in the tumourigenesis and progression of head and neck carcinomas. Accordingly, a difference in the mechanisms of HuR export to the cytoplasm between virus-induced cancers and other cancers has been suggested [21], a hypothesis that makes head and neck tumours suitable candidates for investigating this molecule.

The aim of the present review is to critically summarise the role of HuR in head and neck carcinomas, as presented in the literature, not only in clinical studies but also with *in vitro* experiments or *in vivo* animal models. Initially, we present a comprehensive overview of HuR involvement in the cellular physiology. Subsequently, we summarise HuR expression in cell lines and tissue samples of oral squamous cell carcinoma (OSCC), as well as its premalignant lesions, and discuss its possible significance in terms of clinical course and diagnosis. Additionally, we outline the mechanisms modulating HuR expression, highlighting the subsequent modification of its activity in OSCC. Finally, we describe the current data regarding HuR protein expression and function in the remaining tumour of the head and neck region.

## 2. HuR and Cellular Physiology

**Figure 1:**
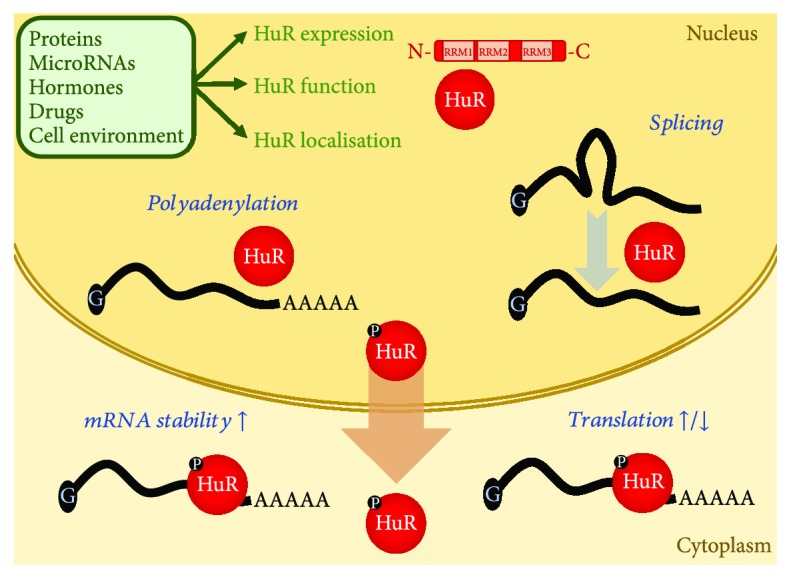
Schematic representation of HuR regulation and function. HuR modulators (proteins, microRNAs, hormones, drugs, and cellular environmental conditions) may affect HuR expression, activity, and subcellular localisation. HuR nucleocytoplasmic shuttling is controlled *via* posttranslational modifications (e.g., phosphorylation). HuR binds to mRNAs through its 3 RNA recognition motifs (RRMs); it has been implicated in splicing and polyadenylation and most importantly in positive regulation of mRNA stability and positive or negative regulation of transcription.

The human *HuR/ELAV1* is located on chromosome 19 at position 19p13.2 [22] and encodes a 32 kD protein, which binds to mRNA targets *via* three highly conserved RNA-binding domains connected by a short-hinge region, belonging to the *RNA recognition motif* (RRM) superfamily [23]; RRM-1 and RRM-2 both bind to elements rich in adenosine/uridine (AU-rich elements, ARE), and RRM-3 binds to the polyadenylate tail of rapidly degrading mRNAs [24]. Similarly, a HuR-binding RNA motif has been recognised, which is a U-rich sequence approximately 17–20 nucleotides in length, mostly located at the 3′ untranslated region (UTR) of the target RNA [25]. Once HuR is connected to its target RNA, the regulation of the stability, translation, and subcellular shuttling of the latter begins [26, 27]. In particular, HuR is reported to stabilise the target mRNA and therefore to indirectly increase the respective protein production [28], whereas its direct effect on translation can be either positive or negative, depending each time on specific function modulators [29–31]. HuR often binds to an internal ribosome entry site (IRES) at the 5′-UTR of cellular mRNAs in order to regulate their translation [29, 32–34], and this is the case for viral RNAs during infection as well [35, 36]. In this context, an interplay between HuR and miRNAs has been recently reported responsible for the expression regulation of specific genes [25, 37–39]. Moreover, HuR appears to modulate mRNA polyadenylation and exon-intron splicing, processes which both take place in the cell nucleus [31, 37]. A schematic representation of HuR regulation and function is illustrated in Figure 1.

Several ARE-containing mRNA targets of HuR have been described, among which cytokines, chemokines and proteins involved in the cell cycle progression, senescence, and inflammation as well as stress response are included [40, 41]. Notably, HuR can stabilise the mRNA, thus increasing the protein expression, of cyclooxygenase-2 (COX-2), an enzyme that catalyses prostaglandin synthesis and is reportedly associated with the promotion of tumourigenesis and tumour angiogenesis [42, 43]. In particular, the proximal region of the 3′-UTR of the *COX-2* gene, which contains several copies of the destabilising motif AUUUA, is the main factor determining the instability of *COX-2* mRNA and is recognised by a multimetric protein complex containing HuR and other RBPs, such as AUF1, TTP, BRF, and KSRP [44–47]. This region regulates the mRNA stability *via* interactions with the sequence-specific RBPs, which influence two steps in eukaryotic decay, deadenylation and/or subsequent 3′ to 5′ degradation of the mRNA [48].

The exact mechanisms involved in the regulation of HuR protein expression and function remain still elusive. A number of HuR modulators at mRNA or protein levels have been reported, among which nitric oxide (NO), 17*β*-estradiol, and foskolin figure prominently [12]. MicroRNAs, including miR-519 [49] and miR-125a [50], have been found to repress HuR translation without affecting HuR mRNA levels, highlighting the importance of measuring directly the abundance of HuR protein in functional and clinical studies. Furthermore, HuR is degraded *via* the ubiquitin-proteasome system and undergoes caspase-mediated cleavage through apoptosis [13, 40]. Importantly, HuR function is reportedly regulated by its subcellular localisation [51]. Under normal healthy conditions, the protein is located in the nucleus but can shuttle to the cytoplasm in order to allow its mRNA target to be processed [52]. This nuclear-cytoplasmic shuttling is achieved through a nuclear-cytoplasmic shuttling sequence (NCS), a 52-amino acid region, located between RRM2 and RRM3, which in association with transportins 1 and 2 (Trn 1 and 2) allows the transportation of the HuR protein, along with the bound mRNA, through the nuclear pores to the cytoplasm [52]. The subcellular shuttling of HuR protein is regulated by several endogenous or exogenous stimuli, such as insulin or DNA damage [53, 54]. In addition, many signalling pathways, including mitogen-activated protein kinases (MAPKs) or members of the protein kinase C (PKC) family, have been recognised to be involved in the modulation of HuR localisation within the cell, in some cases, by inducing the phosphorylation of HuR within the region that contains the NCS sequence [55, 56]. In the same context, there is recent evidence that HuR methylation may play a similar role [57]. Furthermore, several proteins, such as SETalpha, SETbeta, pp32, and acidic protein rich in leucine (APRIL), appear to bind to specific HuR regions, thus modifying its ability to translocate to the cytoplasm [58, 59]. Both pp32 and APRIL contain leukine-rich domains homologous to nuclear export signals known to interact with CRM1 (chromosomal region maintenance protein 1), the nuclear export receptor for the HIV-1 Rev protein [60]. These data suggest that the export of HuR to the cytoplasm might occur by at least two different pathways; one being CRM1-dependent and involving its protein ligands, while the other is CRM-1 independent and requires its endogenous shuttling signal NCS [61]. For example, it has been suggested that in the adenovirus-transformed cells, HuR translocation to the cytoplasm is performed in a CRM1-independent manner, whereas during heat shock stimulation, the HuR shuttling is CRM1-dependent [61, 62].

## 3. HuR in Oral Squamous Cell Carcinoma (OSCC)

### 3.1. HuR Expression in OSCC Cell Lines (Table 1)

HuR expression has been repeatedly investigated in a variety of oral cancer cell lines. Among these, YD9, Y10B, Y32, and Y38, which are human OSCCs, figure prominently [63–65]. Additionally, HSC2 established from an OSCC located on the floor of the mouth, HSC3 established from a SCC located on the tongue, and Ca9-22 established from a gingival SCC were also frequently studied [21, 63, 64, 66], while the oral cancer cell line UM74B was used less frequently [67].

Immunoblot analysis in all the above cell lines showed that HuR is abundantly located in the cytoplasm [21, 63–66], whereas in some investigations, a predominantly cytoplasmic HuR protein expression was reported [63, 64]. The cytoplasmic localisation of HuR was confirmed by immunoblotting on nuclear and cytoplasmic fractions separately. Conversely, in normal gingival fibroblasts and periodontal ligament cells, HuR protein was located only in the cell nucleus, as reported by Hasegawa et al. [66]. Accordingly, HuR mRNA levels have also been assessed by reverse transcriptase-polymerase chain reaction (RT-PCR) [64]. No significant variation of the protein or mRNA expression levels of HuR among these cell lines has been reported, although probable lower HuR mRNA levels could be hypothesised in the Ca9-22 cell line, as observed by Cha et al. [64].

### 3.2. HuR Expression in OSCCs and Premalignant Lesions (Table 2)

Numerous studies have revealed the presence of HuR in the cytoplasm of OSCC tissue samples, ranging from 60 to 71.6% of the investigated cases [63, 64, 66, 68]. Nevertheless, the nuclear expression of HuR was higher, ranging from 91 to 93.2% of the investigated cases [63, 64, 68]. The adjacent nontumour squamous epithelium repeatedly showed solely nuclear HuR immunostaining [63, 64, 66, 67]. In the same context, oral verrucous carcinomas almost always display cytoplasmic HuR immunoreactivity (100%, 17/17 investigated cases in Habiba et al. [69]).

Moreover, HuR is expressed in oral preneoplastic lesions in 55% of the cases and is mainly detected in the nuclei of epithelial cells, whereas cytoplasmic expression is rarely noted [69]. Furthermore, HuR localisation appears to be significantly associated with the level of dysplasia. In particular, according to Habiba et al. [70], in the majority of the low-grade dysplasia cases (76%, 13/17), HuR was either not expressed or expressed in the lower third of the epithelium, whereas most of the high-grade dysplasia cases (71%, 24/34) demonstrated HuR expression either in the lower two-thirds or extending to the upper one-third of the epithelium. Similar observations have been reported for oral verrucous premalignant lesions, such as oral verrucous hyperplasia (OVH) and oral verrucous borderline lesions (OVL) [69]. The latter is defined as epithelial hyperplasia with hyperkeratosis and a verrucous surface, noninvasion of the hyperplastic epithelium into the lamina propria with adjacent normal mucosal epithelium, and lesions with varying degrees of epithelial dysplasia [69]. In all OVH cases, HuR was restricted to the lower one-third of the epithelium and there was a general trend for a more diffuse staining pattern throughout the epithelium in OVCs compared to OVH and OVLs [69]. Additionally, the mean labelling index (LI) of HuR in OVCs was 42.7-fold higher than in OVHs and 2.4-fold higher than in OVLs [69]. Interestingly, HuR expression in premalignant lesions appears to be a good indicator of malignant transformation. Patients with low- or high-grade oral squamous epithelial dysplasia demonstrating HuR expression experienced a significantly increased oral cancer incidence and a shorter time to malignant transformation when compared to patients that did not express the protein (4.99-fold increased risk of malignant transformation) [70]. Accordingly, OVL cases with high HuR expression (defined as >27%) mostly showed expression in the lower two-thirds of the epithelium (90%) and 60% of the cases underwent malignant transformation within 3 years, whereas none of the cases with a low HuR LI (defined as ≤27%) displayed malignant transformation [69]. Acknowledging the substantial interobserver and intraobserver variation in terms of evaluating the presence and severity of epithelial dysplasia [71, 72], these data suggest that HuR could be possibly used as an additional biomarker for evaluating malignant transformation risk in oral premalignancy.

### 3.3. Clinical Significance of HuR Expression in OSCCs

Apart from being correlated with a malignant phenotype, cytoplasmic HuR expression has also been associated with parameters representing a more aggressive tumour behaviour, that is, histological grade [63, 64] as well as the presence of lymph node [63, 68] and distant metastasis [63]. In the light of the above observations, it is not unexpected that cytoplasmic HuR expression has also been correlated in two studies with patient adverse overall survival [63, 64]. This association remained in both studies significant in multivariate survival analysis, indicating cytoplasmic HuR expression as an adverse prognosticator in OSCCs, independent of common prognostic factors, such as histological grade and presence of lymph node or distant metastasis [63, 64]. In contrast, Kim et al. did not manage to establish a significant correlation between cytoplasmic HuR expression and patient prognosis either in univariate or in multivariate survival analysis [68]. Nuclear HuR expression repeatedly does not convey any significant prognostic information in this regard [63, 64, 68].

### 3.4. Modulation of HuR Expression in OSCCs

Several studies have investigated the modulation of HuR expression or activity in OSCCs, as well as its ability to regulate different biological processes. Transfection of YD10B, Ca9-22, and HSC3 cell lines by small interfering RNAs (siRNAs) [21, 63, 64] or short hairpin RNAs (shRNAs) [65] resulted in reduction of cytoplasmic HuR expression, as shown by immunoblotting. Cha et al. in both studies [63, 64] demonstrated a HuR knockdown in YD10B and HSC3 cell lines after treatment with Leptomycin B (LMB), which inhibits the transport of HuR-binding proteins from nucleus to the cytoplasm. In contrast, Hasegawa et al. [66] failed to observe an inhibition of the accumulation of HuR in the cytoplasm of HSC3 and Ca9-22 cell lines after 7 h of treatment with LMB, suggesting that in OSCCs, HuR is exported to the cytoplasm in a manner different from that of normal cells (CRM1 independent). Keeping in mind that in the former two studies, HuR knockdown by LMB was induced after 24 h of treatment and that Ca9-22 cell line is reported to be partially contaminated with MSK9-22 [73]; further studies are essential to determine the exact modulation effect of LMB on HuR subcellular localisation and subsequent role.

Additionally, KPS-A (3-0-[L-rhamnopzranosyl-(1➞2)-*α*-L-arabinopyranosyl]hederagenin), an oleanane triterpene saponin, has been shown to downregulate cytoplasmic HuR levels in YD10B cells [65]. KPS-A has been reported to have several cytotoxic effects in numerous types of cancer cells [74] and to inhibit the growth of colon and lung carcinomas in mice [75, 76]. Moreover, KPS-A was able to restore the nuclear levels of HuR to the control levels in a dose-related manner in YD10B cells stimulated with PMA, a well-known inflammatory stimulator and tumour promoter [65]. Interestingly, the study of Hwang et al. [65] suggests that KPS-A controls HuR expression *via* regulating PI3K/AKT and/or ERK activation.

Recently, the influence of hypoxia in the expression and subcellular localisation of HuR in OSCCs has been investigated [67]. In the study of Talwar et al. [67], it is shown that chronic hypoxic treatment (CoCL_2_ for >8 h) of UM74B OSCC cells induces HuR export to the cytoplasm and its capsase-mediated cleavage. Moreover, the authors suggest a model in which a portion of HuR in OSCCs is cleaved during hypoxia, generating the HuR-cleavage product 1 (HuR-CP1), which strongly interacts with ARE-containing mRNAs, thus promoting their stability and controlling their translation in OSCCs [67].

### 3.5. HuR Activity in OSCCs

HuR protein has been recently reported to have a significant role in tumour angiogenesis, mainly supported by its association with the upregulation of VEGF-A and COX-2 in tumour endothelial cells, thus keeping an angiogenic switch on and activating angiogenic phenotype [13]. This effect is attributed to the fact that the mRNAs transcribed from *VEGF-A* and *COX-2* genes include AU-rich elements and can be stabilised by HuR protein [77]. Cytoplasmic HuR expression is also associated with COX-2 expression in breast, ovarian, gastric, and colorectal cancers and is known to be a poor prognostic variable in these malignancies [63, 78–81]. In keeping with these findings, the LMB-mediated inhibition of cytoplasmic HuR expression in YD10B and HSC-3 OSCC cells has been found to suppress COX-2 expression [63]. Similar results have been reported in monocytes as well as in breast, prostate, ovarian, and colon cancer cells [78, 82, 83]. A possible explanation for this observation is that LMB inhibits the nucleocytoplasmic transport of HuR protein/COX-2 mRNA complexes [63]. The effect of HuR protein on *COX-2* mRNA stabilisation has also been demonstrated in OSCC cell lines treated with siRNAs [21, 63]. In particular, when transcription was blocked with actinomycin D, the levels of *COX-2* mRNA decreased faster in HuR siRNA-treated than in untreated oral cancer cells [63].

HuR knockdown either by LMB or by siRNAs in YD10B and HSC3 cell lines showed that HuR plays a significant role in the regulation of cell apoptosis in OSCCs, as demonstrated by immunoblotting, which revealed a concentration-dependent suppression of cIAP2 (BIRC3) cytoplasmic expression [64]. This protein belongs to the human inhibitor of apoptosis (IAP) family, is characterised by the presence of the baculoviral IAP repeat, zinc ring finger, and caspase recruitment, and inhibits active caspase-3 and caspase-7 directly and activation of procaspase-9 [84, 85]. The mRNA of IAP2 protein belongs to group 3 of ARE proteins, containing 3 pentameric AUUUA repeats [86]. The Bcl-2 mRNA contains the same group 3 AREs as cIAP2 mRNA and binding of HuR is reported to modulate Bcl-2 mRNA stability in HL60 acute myeloid leukemia cells and A431 epidermoid carcinoma cells [87]. The significant role of HuR in the regulation of cell apoptosis in OSCCs has been also demonstrated by Talwar et al. [67], who concluded that the depletion of HuR significantly reduces apoptosis.

In addition, modulation of HuR expression is reported to play a key role in the regulation of OSCC invasiveness, as demonstrated by the reduction of the MMP-9 (metalloproteinase-9) levels in the shRNAs-mediated HuR knockdown YD10B cell culture by Hwang et al. [65]. MMP-9, also known as gelatinase-B and 92 kDa type IV collagenase, is responsible as other metalloproteinases for the degradation of the environmental barriers, such as extracellular matrix and basement membrane, and is reportedly involved in the oral cancer invasion process [88–90]. MMP activation is tightly regulated at the transcriptional and the posttranscriptional level and by TIMPs (tissue inhibitor of metalloproteinases), whereas their excessive extracellular activity in tumour cells induces the remodelling of basement membrane, thus influencing the early stages of tumour initiation, growth, invasion, metastasis, and angiogenesis [91]. In the study of Hwang et al. [65], KPS-A also reduced the MMP-9-mediated invasion of PMA-stimulated OSCC cells, by controlling HuR expression *via* ERK and PI3K/AKT activation. Moreover, the oral administration of KPS-A in mice inoculated with YD10B OSCC cells led to substantial inhibition of tumour growth and the expression of HuR, MMP-9, and TIMP-1 [65]. Similar observations regarding the effect of HuR knockdown on invasive activities of OSCC cells have been reported by the study of Kakuguchi et al. [21], in which the average invasion rate of Ca9.22 cells decreased substantially after 24 h transfection with siRNAs, as shown by a Matrigel invasion assay. In the same study, HuR knockdown cells failed to make colonies in soft agar, suggesting that the cells had lost their ability for anchorage-independent cell growth.

A recent study suggests that HuR has the potential to change the characteristics of OSCC cells, at least in part, by affecting their cell cycle [21]. In this study, the expression of cell cycle-related proteins, such as cyclin A, cyclin B1, cyclin D1, and cyclin-dependent kinase 1 (CDK1), was reduced in HuR knockdown HSC-3 and Ca9.22 cells, whereas HuR was proven to bind to CDK1 mRNA in order to stabilise it [21]. A senescent phenotype in these cells was confirmed by the absence of senescence-associated reporter activity. Cyclin A, cyclin B1, and cyclin D1 mRNAs have been previously recognised as HuR regulated [53, 92, 93]. CDK1 has been shown to be essential [94, 95] and important for the import of HuR to the nucleus, due to its phosphorylation at residue 202 [96]. Importantly, the presence of a feedback loop between the HuR phosphorylation and CDK1 synthesis has been hypothesised [21].

A key role in the regulation of protooncogenes, such as *c-fos* and *c-myc*, has also been attributed to HuR [21, 66, 67]. Both c-fos and c-myc mRNAs contain AREs and were detected in both the nucleus and the cytoplasm of the HSC3 and Ca9-22 cells, but only in the nucleus in normal gingival fibroblast and periodontal ligament cells, as confirmed by in situ hybridisation [66]. These mRNAs had a longer half-time in HSC3 and Ca9-22 and accumulated in higher quantities compared to normal cells, an observation indicating their stabilisation in OSCCs [66]. Moreover, HuR knockdown through siRNAs in oral cancer cells reduced the export and accumulation of *c-myc* mRNA [66]. Another recent study reported that the cytoplasmic expression of *c-fos* and *c-myc* mRNAs was inhibited in the HuR knockdown cells, compared to control cells that had not been transfected with a siRNA, and the half-lives of these mRNAs were shorter than those of their counterparts in the control cells [21]. The HuR-mediated regulation of c-myc mRNA is also demonstrated in the study of Talwar et al. [67], in which HuR-CP1 was found to strongly associate with the 3′-UTR of *c-myc* mRNA and block its mRNA translation in UM74B cells during CoCL_2_-induced hypoxic stress. This interaction was confirmed using ribonucleoprotein immunoprecipitation and site-directed mutagenesis at the AU-rich element sequences of the c-myc mRNA [67]. Surprisingly, siRNA knockdown of HuR elevated c-myc protein expression under hypoxia [67].

## 4. HuR in Other Head and Neck Carcinomas

Although HuR in OSCCs has been investigated by a variety of studies, the currently existing data regarding its expression, modulation, and activity or its correlation with clinicopathological features in the remaining head and neck tumours is rather limited. A presentation of the respective data will follow (Tables 1 and 2).

### 4.1. Thyroid Lesions

HuR expression has been investigated in 8 different thyroid cell lines: Nthy-ori-3.1, derived from normal thyroid follicular epithelial cells; BCPAP; K1; TPC1, derived from papillary thyroid carcinoma (PTC); FTC133; WRO, derived from follicular thyroid carcinoma (FTC); FRO; and SW1736, derived from anaplastic thyroid cancer (ATC) [97]. A significant overexpression of HuR protein was detected in all PTCs and in SW1736 cells, according to immunoblot analysis, whereas HuR positivity was higher in BCPAP compared to Nthy-ori-3.1 cells as shown by immunocytochemistry [97].

HuR expression has been noted in the majority of tissues from benign and malignant thyroid lesions, that is, hyperplastic nodules, Hashimoto thyroiditis, follicular adenomas, FTCs, PTCs, and ATCs, with a moderate to high immunoreactivity in almost half of those [97, 98]. Normal thyroid tissue was negative for HuR immunostaining or showed lower expression compared to tumour lesions [97, 98]. Cytoplasmic HuR immunostaining appears to clearly distinguish not only between normal and tumour tissue but also malignant and benign neoplasia. In particular, cytoplasmic HuR expression is higher in malignant lesions [97, 98], with the highest levels being observed in the group of papillary thyroid carcinomas [97]. These data indicate that HuR may be translocated from nucleus to cytoplasm during the malignant thyroid transformation process.

HuR silencing through siRNAs reduced cell viability in both BCPAP and Nthy-ori-3.1 cell lines, increasing the percentage of apoptotic cells, an observation that indicates a positive role of HuR in cell proliferation in thyroid tissue [97]. In line with this finding, elevated HuR immunoreactivity in thyroid tissue has been associated with increased follicular cells' proliferation rate, as indicated by Ki-67 immunopositivity [98]. Regarding the association of HuR protein with clinicopathological characteristics of thyroid carcinomas, a trend of correlation with the presence of lymphatic invasion has also been noted [98].

Furthermore, global transcriptome analysis has indicated that HuR knockdown *via* siRNA induces distinct gene expression modifications in BCPAP and Nthy-ori-3.1 cell lines [97]. In particular, 807 genes were differentially expressed after HuR silencing in Nthy-ory-3.1 (437 upregulated and 370 downregulated) while, in BCPAP, the differentially expressed genes were 404 (273 upregulated and 131 downregulated) [97]. Only 67 and 29 among the upregulated and the downregulated genes, respectively, were modified in both cell lines [97]. Interestingly, the majority of the modified genes after HuR silencing belongs to the noncoding transcript family, in particular miRNAs [97]. Moreover, the HuR-bound RNA profiles, as evaluated by the RIP-seq approach, appear to be distinct among BCPAP, K1, TPC1, and Nthy-ori-3.1 cell lines, with a set of 114 HuR-bound RNAs distinguishing tumorigenic cell lines from the nontumorigenic one [97]. Among the interesting HuR targets reported, *eIF4E*, *BCL2*, *TP53*, *XIAP*, *MDM2*, *VHL*, and *MYC* are included [97].

The only HuR target whose association with HuR in thyroid lesions has been investigated is COX-2. In the study of Giaginis et al. [98], one-third of the thyroid lesions showed concomitant moderate/high HuR/COX-2 expression, a finding which was more frequently observed in malignant compared to benign thyroid lesions, as well as in PTCs compared to hyperplastic nodules and FTCs. Moreover, concurrent high HuR/COX-2 expression was associated with an increased proliferation index of follicular cells, as measured by Ki-67 staining. In the same study, a positive association between HuR and COX-2 expression was established, which appeared to be stronger in the subgroup of benign lesions [98]. This coexpression of HuR and COX-2, mostly noted in benign lesions, could suggest that the cooperation of these molecules may be biologically more important in benign premalignant conditions when inflammation also plays a crucial role.

### 4.2. Laryngeal Squamous Cell Carcinomas (LSCCs)

According to Cho et al. [44], the nuclear and cytoplasmic HuR expression is significantly higher in the laryngeal carcinomas than in normal and dysplastic laryngeal epithelium. In particular, high nuclear HuR staining was observed in all (39/39) laryngeal squamous cell carcinomas (LSCCs), in the majority (90%, 27/30) of the cases with laryngeal epithelial dysplasia and in half (19/38) of the specimens with a normal-appearing laryngeal epithelium [44]. In addition, cytoplasmic HuR staining was observed in 26 of 39 (66.6%) LSCCs, in one of 30 (3.3%) lesions with epithelial dysplasia and none (0/38) of the specimens with a normal-appearing laryngeal epithelium. However, cytoplasmic HuR expression was not significantly associated with any of the clinicopathological characteristics including histological grade [44]. These findings support the involvement of HuR in laryngeal carcinogenesis and further indicate that cytoplasmic HuR expression could be used to determine the degree of malignant behaviour in laryngeal biopsies, particularly in those of a borderline nature.

Moreover, a significant correlation between high COX-2 immunoreactivity and cytoplasmic HuR expression in LSCCs has been documented, further advocating the significant role of HuR in the regulation of COX-2 in LSCCs [44]. Indeed, among the 26 cases of LSCCs showing high cytoplasmic HuR immunoreactivity, 22 cases (84.6%) showed high expression of COX-2 and only four cases (15.3%) displayed low or no COX-2 immunoreactivity [44].

### 4.3. Salivary Gland Tumours

Regarding normal salivary gland tissue, HuR expression has been demonstrated in A5 and HSG cell lines (derived from rat and human submandibular gland, resp.) by immunoblot and immunofluorescence, similarly to tissue samples of rat submandibular and human parotid glands [99]. Moreover, HuR expression has been investigated in a single study on human pleomorphic adenoma and mucoepidermoid carcinoma, the most common benign and malignant neoplasia of the salivary glands, respectively [100]. In this study, the frequency of HuR cytoplasmic positivity was higher in the mucoepidermoid carcinomas than in the pleomorphic adenomas (35.7% in pleomorphic adenomas versus 72.2% in mucoepidermoid carcinomas). Although the level of nuclear HuR expression was similar among the specific cell types of pleomorphic adenoma and mucoepidermoid carcinoma, cytoplasmic HuR expression was higher in the epidermoid cells than in the mucous cells of mucoepidermoid carcinoma [100]. A statistically significant correlation between the level of cytoplasmic HuR expression and histological grade of mucoepidermoid carcinoma was not established. Furthermore, the authors demonstrated a positive correlation between COX-2 immunoreactivity and cytoplasmic HuR expression in mucoepidermoid carcinomas, but not in pleomorphic adenomas [100].

Experimental data have also shown that transfection of A5 and HSG cell lines with a reporter plasmid carrying the p53 HuR protein-binding site resulted in high luciferase activity in salivary cells. Similar results were observed *in vivo* with transfection of rat submandibular glands [99]. Moreover, inhibition of HuR protein activity by shRNAs in A5 cells demonstrated that this high luciferase activity was mediated by the interaction between HuR protein and the p53 HuR protein-binding site [99]. These findings also emphasise the key role of HuR in the regulation of target proteins in salivary glands.

### 4.4. Oesophageal Squamous Epithelial Cells (OESECs)

Donahue et al. [101] recently investigated HuR in a human OESEC cell line, derived from human oesophageal specimens harvested at the time of donor lung procurement. The authors demonstrated the binding of HuR to a 288 bp fragment in the 3′-UTR of survivin mRNA through specific binding sites in these cells [101]. Surprisingly, overexpression of HuR, which was conducted through infection with recombinant adenoviral vectors, resulted in a decrease of survivin expression and was associated with decreased survivin mRNA and promoter activity, suggesting a decrease in survivin transcription [101]. Concomitantly, the levels of p53, which is considered to be a negative transcriptional regulator of survivin, increased following HuR overexpression, in conjunction with enhanced p53 mRNA stability [101]. This observation suggests that the decrease of survivin transcription, following HuR overexpression, is probably related to the increase of p53 protein. Interestingly, p53 silencing before HuR overexpression promoted the mRNA stability and protein expression of survivin [101]. This finding implies that the role of HuR in the regulation of survivin transcription and stabilisation is influenced by the interaction between p53 and survivin in human OESECs. Similar observations have been reported in breast carcinomas [102].

## 5. Conclusion

HuR protein is expressed in the majority of the cases in all the tumours of the head and neck region examined. More importantly, higher levels of HuR expression have been noted in malignant lesions, such as OSCCs, when compared to normal cells, a difference which is more significant in terms of cytoplasmic HuR expression [63, 64, 68] and is demonstrated not only in tissue samples but also in cell lines, in which cytoplasmic localisation of HuR was confirmed by immunoblotting separately nuclear and cytoplasmic fractions. The same observation was made when comparing malignant tumours with either benign tumours (i.e., thyroid carcinomas versus follicular adenoma, mucoepidermoid carcinoma versus pleomorphic adenoma) [97, 100] or premalignant lesions (OSCCs versus dysplasia, verrucous carcinoma versus verrucous hyperplasia or verrucous borderline lesions) [69, 70], in which HuR staining pattern has been proposed as an additional diagnostic tool. Another interesting finding is the reported distinct HuR-bound profiles among benign and malignant thyroid cells [97], which indicates the important role of HuR regarding the altered phenotype of the malignant cells at the translational level.

As previously reported, HuR binds to several mRNAs that encode proteins involved in malignant transformation. Thus, it induces their expression through mRNA stabilisation and/or altered translation. Some of these proteins and their expression correlation or interaction with HuR have been studied in the tumours of the head and neck region. The most thoroughly investigated protein is COX-2, which plays a key role in inflammation, carcinogenesis, and angiogenesis and has been shown to positively correlate with HuR in OSCCs, LSCCs, and thyroid lesions, as well as mucoepidermoid carcinomas [44, 63, 98, 100]. In the same context, *in vitro* interaction between the COX-2 mRNA and HuR has also been demonstrated in OSCCs [63]. Moreover, HuR has been shown to be associated with molecules controlling cell apoptosis (i.e., cIAP2 in OSCCs) [64] and cell proliferation or cycle regulation (i.e., Ki-67 index in thyroid tissue, cyclins A, B1, and D1, CDK1 in OSCCs, survivin in human eosophageal epithelial cells) [21, 98, 101]. Furthermore, HuR has also been reported to interact with oncogenes (i.e., c-myc in OSCCs) [66, 67] as well as molecules regulating tumour invasiveness (i.e., MMP-9 in OSCCs) [65].

Interestingly, HuR appears to have a clinical importance in some tumours of the head and neck region. In particular, cytoplasmic HuR levels are correlated with tumour histological grade in OSCCs [63, 64], lymph node and distant metastasis in OSCCs [63, 68], and lymphatic invasion in thyroid carcinomas [98], thus being associated with a more aggressive phenotype. Interestingly, cytoplasmic HuR expression is an adverse prognosticator in OSCCs [63, 64] and remains significant in multivariate survival analysis including histological grade, presence of lymph node, or distant metastasis. However, the clinical significance of HuR in the remaining head and neck tumours except for OSCCs remains elusive.

The data presented in this review support the consistent role of HuR protein in the carcinogenesis and progression of tumours of the head and neck region. However, further studies are warranted to validate and expand the present information, especially on the remaining carcinomas except for OSCCs. Future studies should also be oriented to elucidate possible differences in the role of HuR between HPV^+^ and HPV^−^ SCCs. Keeping in mind that HuR has been recently found to be implicated in chemoresistance mechanisms to therapeutic drugs, such as tamoxifen [103, 104]; strategies to reduce HuR protein levels could be a promising therapeutic approach in controlling tumour progression. To this end, further investigation is required in order to shed light upon the mechanisms of HuR activity in each tumour type.

## Figures and Tables

**Table 1:** HuR expression, modification, and activity in studies investigating cell lines.

Study	Cell lines investigated	HuR expression	HuR modification and activity
*OSCCs*			
Hasegawa et al. [66]	HSC3, Ca9-22	Presence of cytoplasmic	(i) HuR knockdown (*via* siRNAs) ↓ c-myc export and accumulation (ii) LMB after 7 h did not achieve HuR knockdown (iii) s-fos and c-myc stabilisation
Cha et al. [63]	YD9, Y10B, Y32, Y38, HSC2, HSC3, and Ca9-22	Predominantly cytoplasmic	(i) HuR knockdown (*via* LMB and siRNAs) ↓ cytoplasmic HuR and COX-2 induction
Cha et al. [64]	YD9, Y10B, Y32, Y38, HSC2, HSC3, and Ca9-22	Predominantly cytoplasmic	(i) HuR knockdown (*via* LMB and siRNAs) ↓ cytoplasmic HuR and cIAP2 (concentration dependent)
Kakuguchi et al. [21]	HSC3, Ca9-22	High expression	HuR knockdown (*via* siRNAs) (i) ↓ cytoplasmic HuR (ii) ↓ cytoplasmic c-fos, c-myc, and COX-2 (iii) ↓ cyclin A, B1, D1, and CDK1 expression (iv) ↓ average invasion rate of cells (Matrigel invasion assay) (v) ↑ loss of ability for anchorage-independent cell growth
Hwang et al. [65]	YD10B	Presence of expression	(i) HuR knockdown (*via* shRNAs) ↓ cytoplasmic HuR and MMP-9 (ii) KPS-A controls HuR expression *via* ERK and PI3K/AKT activation under hypoxia
Talwar et al. [67]	UM74B	Overexpression of HuR-CP1	(i) HuR-CP1 associates with c-myc mRNA thus ↓ its translation (ii) HuR knockdown (*via* siRNAs) ↑ c-myc expression

*Thyroid lesions*			
Baldan et al. [97]	Nthy-ori-3.1, BCPAP, K1 TPC1, FTC133 WRO, FRO, and SW1736	Overexpression in all PTCs and in SW1736	(i) HuR knockdown (*via* siRNAs) (a) ↑ apoptotic cells (b) ↑ distinct gene expression modifications in BCPAP and Nthy-ori-3.1 cell lines (ii) Different HuR-bound RNA profiles among BCPAP, K1, TPC1 and Nthy-ori-3.1
Human oesophageal epithelial cells			
Donahue et al. [101]	Derived from human specimens		Regulates survivin, in the absence of p53

**Table 2:** HuR expression, localisation, and associations with clinicopathological features and target molecules as well as patients' overall survival in studies investigating tissue samples.

Study	*N*	HuR localisation		Correlations with		
		Nuclear	Cytoplasmic	Clinicopathological features	Other molecules	Patients' overall survival
*OSCCs*						
Cha et al. [64]	95	91.6% (87/95)	71.6% (68/95)	Grade	Nuclear and cytoplasmic with IAP2	Cytoplasmic HuR adverse prognosticator
Cha et al. [63]	103	93.2% (96/103)	69.9% (72/103)	Gender, grade, lymph node, and distant metastasis	Cytoplasmic HuR with COX-2	Cytoplasmic HuR adverse prognosticator
Kim et al. [68]	96	91% (83/96)	60% (54/96)	Lymph node metastasis	—	Not correlated

*LSCCs*						
Cho et al. [44]	39	100% (39/39)	66.6% (26/39)	None	Cytoplasmic HuR with COX-2	**—**

*Thyroid lesions*						
Giaginis et al. [98]	98	Presence in 80% (78/98), higher expression in 43% (42/98)				
Benign	48	Predominantly nuclear, higher expression in 29% (14/48)			(i) Ki-67 in follicular cells (ii) COX-2 (stronger in benign)	—
Malignant	50	Predominantly cytoplasmic, higher expression in 56% (28/50)		Lymphatic invasion (trend)		—
Baldan et al. [97]	104					
Normal samples	12	(i) ↑ nuclear in all tumours (ii) ↑ cytoplasmic in nontumour tissues versus FAs or PTCs, FTCs and ATCs		—	—	—
Follicular adenomas	25			—	—	—
Carcinomas (PTC, FTC, and ATC)	67			—	—	—

*Salivary gland tumours*						
Cho et al. [100]	46					
Pleomorphic adenoma	28	53.6% (15/28)	35.7% (10/28)	—	—	—
Mucoepidermoid carcinoma	18	77.78% (14/18)	72.22% (13/18)	—	Cytoplasmic HuR with COX-2	—

## References

[1] Audic Y., Hartley R. (2004). Post-transcriptional regulation in cancer. *Biology of the Cell*.

[2] Siomi H., Dreyfuss G. (1997). RNA-binding proteins as regulators of gene expression. *Current Opinion in Genetics & Development*.

[3] Wurth L. (2012). Versatility of RNA-binding proteins in cancer. *Comparative and Functional Genomics*.

[4] Dalmau J., Furneaux H. M., Gralla R. J., Kris M. G., Posner J. B. (1990). Detection of the anti-Hu antibody in serum of patients with small cell lung cancer-a quantitative western blot analysis. *Annals of Neurology*.

[5] Ma W. J., Cheng S., Campbell C., Wright A., Furneaux H. (1996). Cloning and characterization of HuR, a ubiquitously expressed Elav-like protein. *The Journal of Biological Chemistry*.

[6] Farber E. (1984). The multistep nature of cancer development. *Cancer Research*.

[7] Licitra L., Bernier J., Grandi C. (2003). Cancer of the larynx. *Critical Reviews in Oncology/Hematology*.

[8] Cabilla J. P., Nudler S. I., Ronchetti S. A., Quinteros F. A., Lasaga M., Duvilanski B. H. (2011). Nitric oxide-sensitive guanylyl cyclase is differentially regulated by nuclear and non-nuclear estrogen pathways in anterior pituitary gland. *PLoS One*.

[9] Woo H. H., Zhou Y., Yi X. (2009). Regulation of non-AU-rich element containing c-fms proto-oncogene expression by HuR in breast cancer. *Oncogene*.

[10] Calaluce R., Gubin M. M., Davis J. W. (2010). The RNA binding protein HuR differentially regulates unique subsets of mRNAs in estrogen receptor negative and estrogen receptor positive breast cancer. *BMC Cancer*.

[11] Kotta-Loizou I., Giaginis C., Theocharis S. (2014). Clinical significance of HuR expression in human malignancy. *Medical Oncology*.

[12] Kotta-Loizou I., Vasilopoulos S. N., Coutts R. H., Theocharis S. (2016). Current evidence and future perspectives on HuR and breast cancer development, prognosis, and treatment. *Neoplasia*.

[13] Kurosu T., Ohga N., Hida Y. (2011). HuR keeps an angiogenic switch on by stabilising mRNA of VEGF and COX-2 in tumour endothelium. *British Journal of Cancer*.

[14] Siegel R., Ma J., Zou Z., Jemal A. (2014). Cancer statistics, 2014. *CA: A Cancer Journal for Clinicians*.

[15] Müller S. (2017). Update from the 4th edition of the World Health Organization of head and neck tumours: tumours of the oral cavity and mobile tongue. *Head and Neck Pathology*.

[16] Gale N., Poljak M., Zidar N. (2017). Update from the 4th edition of the World Health Organization classification of head and neck tumours: what is new in the 2017 WHO blue book for tumours of the hypopharynx, larynx, trachea and parapharyngeal space. *Head and Neck Pathology*.

[17] Stelow E. B., Wenig B. M. (2017). Update from the 4th edition of the World Health Organization classification of head and neck tumours: nasopharynx. *Head and Neck Pathology*.

[18] Stelow E. B., Bishop J. A. (2017). Update from the 4th edition of the World Health Organization classification of head and neck tumours: tumors of the nasal cavity, paranasal sinuses and skull base. *Head and Neck Pathology*.

[19] Westra W. H., Lewis J. S. (2017). Update from the 4th edition of the World Health Organization classification of head and neck tumours: oropharynx. *Head and Neck Pathology*.

[20] Katabi N., Lewis J. S. (2017). Update from the 4th edition of the World Health Organization classification of head and neck tumours: what is new in the 2017 WHO blue book for tumors and tumor-like lesions of the neck and lymph nodes. *Head and Neck Pathology*.

[21] Kakuguchi W., Kitamura T., Kuroshima T. (2010). HuR knockdown changes the oncogenic potential of oral cancer cells. *Molecular Cancer Research*.

[22] Ma W. J., Furneaux H. (1996). Localization of the human HuR gene to chromosome 19p13.2. *Human Genetics*.

[23] Burd C. G., Dreyfuss G. (1994). Conserved structures and diversity of functions of RNA-binding proteins. *Science*.

[24] Govindaraju S., Lee B. S. (2013). Adaptive and maladaptive expression of the mRNA regulatory protein HuR. *World Journal of Biological Chemistry*.

[25] Fan X. C., Steitz J. A. (1998). HNS, a nuclear-cytoplasmic shuttling sequence in HuR. *Proceedings of the National Academy of Sciences of the United States of America*.

[26] Myer V. E., Fan X. C., Steitz J. A. (1997). Identification of HuR as a protein implicated in AUUUA-mediated mRNA decay. *The EMBO Journal*.

[27] Fan X. C., Steitz J. A. (1998). Overexpression of HuR, a nuclear-cytoplasmic shuttling protein, increases the *in vivo* stability of ARE-containing mRNAs. *The EMBO Journal*.

[28] Peng S. S., Chen C. Y., Xu N., Shyu A. B. (1998). RNA stabilization by the AU-rich element binding protein, HuR, an ELAV protein. *The EMBO Journal*.

[29] Kullmann M., Gopfert U., Siewe B., Hengst L. (2002). ELAV/Hu proteins inhibit p27 translation via an IRES element in the p27 5^'^UTR. *Genes & Development*.

[30] Mazan-Mamczarz K., Galban S., Lopez de Silanes I. (2003). RNA-binding protein HuR enhances p53 translation in response to ultraviolet light irradiation. *Proceedings of the National Academy of Sciences of the United States of America*.

[31] Zhu H., Zhou H. L., Hasman R. A., Lou H. (2007). Hu proteins regulate polyadenylation by blocking sites containing U-rich sequences. *The Journal of Biological Chemistry*.

[32] Meng Z., King P. H., Nabors L. B. (2005). The ELAV RNA-stability factor HuR binds the 5′-untranslated region of the human IGF-IR transcript and differentially represses cap-dependent and IRES-mediated translation. *Nucleic Acids Research*.

[33] Yeh C. H., Hung L. Y., Hsu C. (2008). RNA-binding protein HuR interacts with thrombomodulin 5^'^untranslated region and represses internal ribosome entry site-mediated translation under IL-1*β* treatment. *Molecular Biology of the Cell*.

[34] Durie D., Hatzoglou M., Chakraborty P., Holcik M. (2013). HuR controls mitochondrial morphology through the regulation of BclxL translation. *Translation*.

[35] Rivas-Aravena A., Ramdohr P., Vallejos M. (2009). The Elav-like protein HuR exerts translational control of viral internal ribosome entry sites. *Virology*.

[36] Lin J. Y., Brewer G., Li M. L. (2015). HuR and Ago2 bind the internal ribosome entry site of enterovirus 71 and promote virus translation and replication. *PLoS One*.

[37] Izquierdo J. M. (2008). Hu antigen R (HuR) functions as an alternative pre-mRNA splicing regulator of Fas apoptosis-promoting receptor on exon definition. *Journal of Biological Chemistry*.

[38] Mukherjee N., Corcoran D. L., Nusbaum J. D. (2011). Integrative regulatory mapping indicates that the RNA-binding protein HuR couples pre-mRNA processing and mRNA stability. *Molecular Cell*.

[39] Akaike Y., Masuda K., Kuwano Y. (2014). HuR regulates alternative splicing of the *TRA2β* gene in human colon cancer cells under oxidative stress. *Molecular and Cellular Biology*.

[40] Abdelmohsen K., Lal A., Kim H. H., Gorospe M. (2007). Posttranscriptional orchestration of an anti-apoptotic program by HuR. *Cell Cycle*.

[41] Yiakouvaki A., Dimitriou M., Karakasiliotis I., Eftychi C., Theocharis S., Kontoyiannis D. L. (2012). Myeloid cell expression of the RNA-binding protein HuR protects mice from pathologic inflammation and colorectal carcinogenesis. *The Journal of Clinical Investigation*.

[42] Khan Z., Khan N., Tiwari R. P., Sah N. K., Prasad G. B., Bisen P. S. (2011). Biology of Cox-2: an application in cancer therapeutics. *Current Drug Targets*.

[43] Ghosh N., Chaki R., Mandal V., Mandal S. C. (2010). COX-2 as a target for cancer chemotherapy. *Pharmacological Reports*.

[44] Cho N. P., Han H. S., Soh Y., Lee K. Y., Son H. J. (2007). Cytoplasmic HuR over-expression is associated with increased cyclooxygenase-2 expression in laryngeal squamous cell carcinomas. *Pathology*.

[45] Dean J. L., Sully G., Wait R., Rawlinson L., Clark A. R., Saklatvala J. (2002). Identification of a novel AU-rich-element-binding protein which is related to AUF1. *The Biochemical Journal*.

[46] Dixon D. A., Kaplan C. D., McIntyre T. M., Zimmerman G. A., Prescott S. M. (2000). Post-transcriptional control of cyclooxygenase-2 gene expression. The role of the 3′-untranslated region. *The Journal of Biological Chemistry*.

[47] Mazan-Mamczarz K., Gartenhaus R. B. (2007). Post-transcriptional control of the MCT-1-associated protein DENR/DRP by RNA-binding protein AUF1. *Cancer Genomics & Proteomics*.

[48] Nabors L. B., Gillespie G. Y., Harkins L., King P. H. (2001). HuR, a RNA stability factor, is expressed in malignant brain tumors and binds to adenine- and uridine-rich elements within the 3′ untranslated regions of cytokine and angiogenic factor mRNAs. *Cancer Research*.

[49] Abdelmohsen K., Srikantan S., Kuwano Y., Gorospe M. (2008). miR-519 reduces cell proliferation by lowering RNA-binding protein HuR levels. *Proceedings of the National Academy of Sciences of the United States of America*.

[50] Guo X., Wu Y., Hartley R. S. (2009). MicroRNA-125a represses cell growth by targeting HuR in breast cancer. *RNA Biology*.

[51] Katsanou V., Papadaki O., Milatos S. (2005). HuR as a negative posttranscriptional modulator in inflammation. *Molecular Cell*.

[52] Sengupta S., Jang B. C., MT W., Paik J. H., Furneaux H., Hla T. (2003). The RNA-binding protein HuR regulates the expression of cyclooxygenase-2. *The Journal of Biological Chemistry*.

[53] Wang W., Caldwell M. C., Lin S., Furneaux H., Gorospe M. (2000). HuR regulates cyclin A and cyclin B1 mRNA stability during cell proliferation. *The EMBO Journal*.

[54] Lafarga V., Cuadrado A., Lopez de Silanes I., Bengoechea R., Fernandez-Capetillo O., Nebreda A. R. (2009). p38 Mitogen-activated protein kinase- and HuR-dependent stabilization of p21^Cip1^ mRNA mediates the G_1_/S checkpoint. *Molecular and Cellular Biology*.

[55] Doller A., Huwiler A., Muller R., Radeke H. H., Pfeilschifter J., Eberhardt W. (2007). Protein kinase C*α*-dependent phosphorylation of the mRNA-stabilizing factor HuR: implications for posttranscriptional regulation of cyclooxygenase-2. *Molecular Biology of the Cell*.

[56] Yoon J. H., Abdelmohsen K., Srikantan S. (2014). Tyrosine phosphorylation of HuR by JAK3 triggers dissociation and degradation of HuR target mRNAs. *Nucleic Acids Research*.

[57] Hinman M. N., Lou H. (2008). Diverse molecular functions of Hu proteins. *Cellular and Molecular Life Sciences*.

[58] Doller A., Winkler C., Azrilian I. (2011). High-constitutive HuR phosphorylation at Ser 318 by PKCδ propagates tumor relevant functions in colon carcinoma cells. *Carcinogenesis*.

[59] Rebane A., Aab A., Steitz J. A. (2004). Transportins 1 and 2 are redundant nuclear import factors for hnRNP A1 and HuR. *RNA*.

[60] Brennan C. M., Gallouzi I. E., Steitz J. A. (2000). Protein ligands to HuR modulate its interaction with target mRNAs in vivo. *The Journal of Cell Biology*.

[61] Gallouzi I. E., Brennan C. M., Steitz J. A. (2001). Protein ligands mediate the CRM1-dependent export of HuR in response to heat shock. *RNA*.

[62] Higashino F., Aoyagi M., Takahashi A. (2005). Adenovirus E4orf6 targets pp32/LANP to control the fate of ARE-containing mRNAs by perturbing the CRM1-dependent mechanism. *The Journal of Cell Biology*.

[63] Cha J. D., Li S., Cha I. H. (2011). Association between expression of embryonic lethal abnormal vision-like protein HuR and cyclooxygenase-2 in oral squamous cell carcinoma. *Head & Neck*.

[64] Cha J. D., Kim H. K., Cha I. H. (2014). Cytoplasmic HuR expression: correlation with cellular inhibitors of apoptosis protein-2 expression and clinicopathologic factors in oral squamous cell carcinoma cells. *Head & Neck*.

[65] Hwang Y. S., Park K. K., Chung W. Y. (2012). Kalopanaxsaponin A inhibits the invasion of human oral squamous cell carcinoma by reducing metalloproteinase-9 mRNA stability and protein trafficking. *Biological and Pharmaceutical Bulletin*.

[66] Hasegawa H., Kakuguchi W., Kuroshima T. (2009). HuR is exported to the cytoplasm in oral cancer cells in a different manner from that of normal cells. *British Journal of Cancer*.

[67] Talwar S., Jin J., Carroll B., Liu A., Gillespie M. B., Palanisamy V. (2011). Caspase-mediated cleavage of RNA-binding protein HuR regulates c-Myc protein expression after hypoxic stress. *Journal of Biological Chemistry*.

[68] Kim K. Y., Li S., Cha J. D., Zhang X., Cha I. H. (2012). Significance of molecular markers in survival prediction of oral squamous cell carcinoma. *Head & Neck*.

[69] Habiba U., Kitamura T., Yanagawa-Matsuda A. (2014). Cytoplasmic expression of HuR may be a valuable diagnostic tool for determining the potential for malignant transformation of oral verrucous borderline lesions. *Oncology Reports*.

[70] Habiba U., Kitamura T., Yanagawa-Matsuda A. (2016). HuR and podoplanin expression is associated with a high risk of malignant transformation in patients with oral preneoplastic lesions. *Oncology Letters*.

[71] Karrabul A., Reibel J., Therkildsen M. H., Praetorius F., Nielsen H. W., Dabelsteen E. (1995). Observer variability in the histologic assessment of oral premalignant lesions. *Journal of Oral Pathology & Medicine*.

[72] Abbey L. M., Kaugars G. E., Gunsolley J. C. (1995). Intraexaminer and interexaminer reliability in the diagnosis of oral epithelial dysplasia. *Oral Surgery, Oral Medicine, Oral Pathology, Oral Radiology*.

[73] Zhao M., Sano D., Pickering C. R. (2011). Assembly and initial characterization of a panel of 85 genomically validated cell lines from diverse head and neck tumor sites. *Clinical Cancer Research*.

[74] Lee K. T., Sohn I. C., Park H. J., Kim D. W., Jung G. O., Park K. Y. (2000). Essential moiety for antimutagenic and cytotoxic activity of hederagenin monodesmosides and bisdesmosides isolated from the stem bark of *Kalopanax pictus*. *Planta Medica*.

[75] Park H. J., Kwon S. H., Lee J. H., Lee K. H., Miyamoto K., Lee K. T. (2001). Kalopanaxsaponin A is a basic saponin structure for the anti-tumor activity of hederagenin monodesmosides. *Planta Medica*.

[76] Kumara S. S., Huat B. T. (2001). Extraction, isolation and characterisation of antitumor principle, *α*-hederin, from the seeds of *Nigella sativa*. *Planta Medica*.

[77] Lopez de Silanes I., Lal A., Gorospe M. (2005). HuR: post-transcriptional paths to malignancy. *RNA Biology*.

[78] Niesporek S., Kristiansen G., Thoma A. (2008). Expression of the ELAV-like protein HuR in human prostate carcinoma is an indicator of disease relapse and linked to COX-2 expression. *International Journal of Oncology*.

[79] Lim S. J., Kim H. J., Kim J. Y., Park K., Lee C. M. (2007). Expression of HuR is associated with increased cyclooxygenase-2 expression in uterine cervical carcinoma. *International Journal of Gynecological Pathology*.

[80] Mrena J., Wiksten J. P., Thiel A. (2005). Cyclooxygenase-2 is an independent prognostic factor in gastric cancer and its expression is regulated by the messenger RNA stability factor HuR. *Clinical Cancer Research*.

[81] Denkert C., Weichert W., Pest S. (2004). Overexpression of the embryonic-lethal abnormal vision-like protein HuR in ovarian carcinoma is a prognostic factor and is associated with increased cyclooxygenase 2 expression. *Cancer Research*.

[82] Erkinheimo T. L., Lassus H., Sivula A. (2003). Cytoplasmic HuR expression correlates with poor outcome and with cyclooxygenase 2 expression in serous ovarian carcinoma. *Cancer Research*.

[83] Jang B. C., Munoz-Najar U., Paik J. H., Claffey K., Yoshida M., Hla T. (2003). Leptomycin B, an inhibitor of the nuclear export receptor CRM1, inhibits COX-2 expression. *The Journal of Biological Chemistry*.

[84] Yang Y. L., Li X. M. (2000). The IAP family: endogenous caspase inhibitors with multiple biological activities. *Cell Research*.

[85] Snipas S. J., Stennicke H. R., Riedl S. (2001). Inhibition of distant caspase homologues by natural caspase inhibitors. *The Biochemical Journal*.

[86] JY L., Schneider R. J. (2004). Tissue distribution of AU-rich mRNA-binding proteins involved in regulation of mRNA decay. *The Journal of Biological Chemistry*.

[87] Ishimaru D., Ramalingam S., Sengupta T. K. (2009). Regulation of Bcl-2 expression by HuR in HL60 leukemia cells and A431 carcinoma cells. *Molecular Cancer Research*.

[88] Liu S. Y., Liu Y. C., Huang W. T., Huang G. C., Su H. J., Lin M. H. (2007). Requirement of MMP-3 in anchorage-independent growth of oral squamous cell carcinomas. *Journal of Oral Pathology & Medicine*.

[89] Vairaktaris E., Serefoglou Z., Yapijakis C. (2007). High gene expression of matrix metalloproteinase-7 is associated with early stages of oral cancer. *Anticancer Research*.

[90] Luukkaa H., Klemi P., Hirsimäki P. (2008). Matrix metalloproteinase (MMP)-1, -9 and -13 as prognostic factors in salivary gland cancer. *Acta Oto-Laryngologica*.

[91] Egeblad M., Werb Z. (2002). New functions for the matrix metalloproteinases in cancer progression. *Nature Reviews Cancer*.

[92] Wang W., Yang X., Cristofalo V. J., Holbrook N. J., Gorospe M. (2001). Loss of HuR is linked to reduced expression of proliferative genes during replicative senescence. *Molecular and Cellular Biology*.

[93] Lal A., Mazan-Mamczarz K., Kawai T., Yang X., Martindale J. L., Gorospe M. (2004). Concurrent versus individual binding of HuR and AUF1 to common labile target mRNAs. *The EMBO Journal*.

[94] Santamaria D., Barriere C., Cerqueira A. (2007). Cdk1 is sufficient to drive the mammalian cell cycle. *Nature*.

[95] Malumbres M., Barbacid M. (2009). Cell cycle, CDKs and cancer: a changing paradigm. *Nature Reviews Cancer*.

[96] Kim H., Abdelmohsen K., Lal A. (2008). Nuclear HuR accumulation through phosphorylation by Cdk1. *Genes & Development*.

[97] Baldan F., Mio C., Allegri L. (2016). Identification of tumorigenesis-related mRNAs associated with RNA-binding protein HuR in thyroid cancer cells. *Oncotarget*.

[98] Giaginis C., Alexandrou P., Delladetsima I. (2016). Clinical significance of Hu-antigen receptor (HuR) and cyclooxygenase-2 (COX-2) expression in human malignant and benign thyroid lesions. *Pathology & Oncology Research*.

[99] Zheng C., Baum B. J. (2012). Including the p53 ELAV-like protein-binding site in vector cassettes enhances transgene expression in rat submandibular gland. *Oral Diseases*.

[100] Cho N. P., Han H. S., Soh Y., Son H. J. (2007). Overexpression of cyclooxygenase-2 correlates with cytoplasmic HuR expression in salivary mucoepidermoid carcinoma but not in pleomorphic adenoma. *Journal of Oral Pathology & Medicine*.

[101] Donahue J. M., Chang E. T., Xiao L. (2011). The RNA-binding protein HuR stabilizes survivin mRNA in human oesophageal epithelial cells. *The Biochemical Journal*.

[102] Vegran F., Boidot R., Oudin C., Defrain C., Rebucci M., Lizard-Nacol S. (2007). Association of p53 gene alterations with the expression of antiapoptotic survivin splice variants in breast cancer. *Oncogene*.

[103] Filippova N., Yang X., Wang Y. (2011). The RNA-binding protein HuR promotes glioma growth and treatment resistance. *Molecular Cancer Research*.

[104] Hostetter C., Licata L. A., Witkiewicz A. (2008). Cytoplasmic accumulation of the RNA binding protein HuR is central to tamoxifen resistance in estrogen receptor positive breast cancer cells. *Cancer Biology & Therapy*.

